# Use of anti-VEGF drugs at the Instituto de la Visión de Montemorelos

**Published:** 2014

**Authors:** Pedro A Gomez Bastar

**Affiliations:** CBM Medical Adviser and Chairman, Instituto de la Vision, Universidad de Montemorelos, Mexico pgomez07@gmail.com

**Figure:**
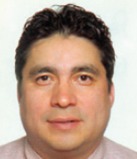
Pedro A Gomez Bastar

## 1 Which anti-VEGF agents do we use?

We use bevazucimab (Avastin) – the dose used is 2.5 mg (0.1 ml). This anti-VEGF agent is used because of its:

- proven efficacy and effectiveness (CATT & IVAN studies)
- low cost, making it affordable for our patients.

**Figure:**
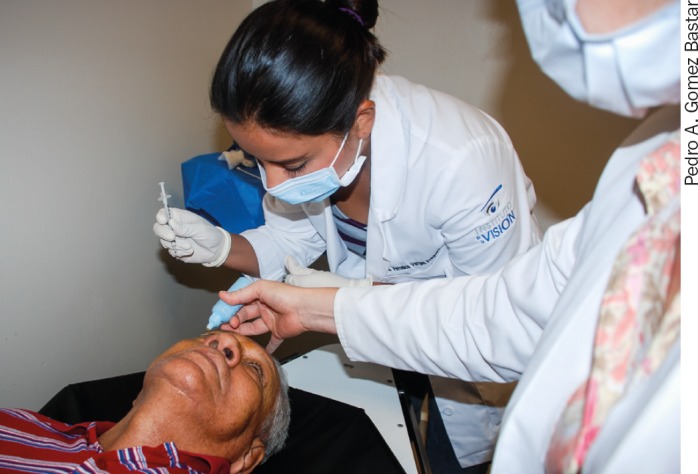
Anti-VEGF injections are given in a clean room

## 2 What are the indications?

- Vitreous haemorrhage secondary to proliferative diabetic retinopathy – particularly when there has been no previous laser.
- Prior to vitrectomy for proliferative diabetic retinopathy.
- Clinically significant macular oedema due to diabetic retinopathy.
- Macular oedema secondary to branch or central retinal vein occlusion.
- Exudative age-related macular degeneration.
- Neovascular glaucoma.

## 3 Who gives the injections?

Intra-vitreal injections are always given by an ophthalmologist, for example:

- retina specialists
- retina subspecialty trainees
- ophthalmology residents in the retinal service.

## 4 Are anti-VEGF agents used without OCT?

Anti VEGF agents are used without OCT in selected cases:

- vitreous haemorrhage secondary to proliferative diabetic retinopathy – particularly when there has been no previous laser
- prior to vitrectomy for proliferative diabetic retinopathy
- clinically significant macular oedema due to diabetic retinopathy
- neovascular glaucoma.

## 5 What are the outcomes?

Clinical experience has been very positive and we believe this is a cost-effective treatment for our patients.

**Vitreous haemorrhage secondary to proliferative diabetic retinopathy:** We have been pleased with our results. Anti-angiogenic therapy reduces the vitreous haemorrhage in many patients with diabetic retinopathy, allowing us to apply laser and avoid vitrectomy surgery.

**Prior to vitrectomy for proliferative diabetic retinopathy:** Application 3–5 days before surgery reduces the risk of intra-operative and postoperative bleeding.

**Neovascular glaucoma:** In these patients, we are careful to avoid further increases in the IOP. When the rubeosis regresses we apply pan-retinal laser, giving us more control of the iris neovascularisation.

**Clinically significant macular oedema:** In clinically significant macular oedema due to diabetic retinopathy, we normally apply three doses of Avastin with 1-month intervals between injections. After the last injection, a macular OCT is requested and, if the oedema has decreased, we apply focal laser.

**Age-related macular degeneration (AMD):** In patients with exudative AMD, an injection is given every month for several months to improve visual acuity and to control the disease, following the ‘treat and extend’ protocol.

